# *PARK* Genes Link Mitochondrial Dysfunction and Alpha-Synuclein Pathology in Sporadic Parkinson’s Disease

**DOI:** 10.3389/fcell.2021.612476

**Published:** 2021-07-06

**Authors:** Wen Li, YuHong Fu, Glenda M. Halliday, Carolyn M. Sue

**Affiliations:** ^1^Brain and Mind Centre, University of Sydney, Sydney, NSW, Australia; ^2^Kolling Institute of Medical Research, Faculty of Medicine and Health, University of Sydney, Royal North Shore Hospital, St Leonards, NSW, Australia; ^3^School of Medical Science, Faculty of Medicine and Health, University of Sydney, Sydney, NSW, Australia

**Keywords:** Parkinson’s disease, mitochondria, mitophagy, α-synuclein pathology, *PARK* genes

## Abstract

Parkinson’s disease (PD) is an age-related neurodegenerative disorder affecting millions of people worldwide. The disease is characterized by the progressive loss of dopaminergic neurons and spread of Lewy pathology (α-synuclein aggregates) in the brain but the pathogenesis remains elusive. PD presents substantial clinical and genetic variability. Although its complex etiology and pathogenesis has hampered the breakthrough in targeting disease modification, recent genetic tools advanced our approaches. As such, mitochondrial dysfunction has been identified as a major pathogenic hub for both familial and sporadic PD. In this review, we summarize the effect of mutations in 11 *PARK* genes (*SNCA, PRKN, PINK1, DJ-1, LRRK2, ATP13A2, PLA2G6, FBXO7, VPS35, CHCHD2*, and *VPS13C*) on mitochondrial function as well as their relevance in the formation of Lewy pathology. Overall, these genes play key roles in mitochondrial homeostatic control (biogenesis and mitophagy) and functions (e.g., energy production and oxidative stress), which may crosstalk with the autophagy pathway, induce proinflammatory immune responses, and increase oxidative stress that facilitate the aggregation of α-synuclein. Thus, rectifying mitochondrial dysregulation represents a promising therapeutic approach for neuroprotection in PD.

## Introduction

Parkinson’s disease (PD) is an age-related neurodegenerative disorder with an insidious onset and a substantial preclinical phase (estimated as >20 years). The progressive aggregation of α-synuclein (Lewy pathology) through the brain and the loss of midbrain dopamine neurons are the pathological landmarks of PD (Surmeier et al., 2017; Fares et al., 2021). Although the etiology of PD is multifactorial, mitochondrial dysfunction has been recognized as a main neuropathogenic mechanism that can affect energy provision and biological pathways (e.g., autophagy, proinflammatory, and antioxidant) to potentially facilitate the Lewy pathology and neuronal loss (Poewe et al., 2017; Park et al., 2018; Grunewald et al., 2019; Pang et al., 2019; Fenton et al., 2021; Onishi et al., 2021).

High-throughput next-generation sequencing and genome-wide association studies have revealed PD risk-associated genes, including 23 *PARK* genes (Schulte and Gasser, 2011; Marras et al., 2017; Poewe et al., 2017) and others (e.g., *HLA-DRA*, *EIF4GI, GBA, MAPT, BSTI, TMEM230, APOE*, and *POLG*). Most *PARK* genes have been revealed as low prevalence (Tran et al., 2020), perhaps a reason of limited research on their involvement in sporadic PD. We reviewed 11 *PARK* genes (Table 1) relevant to mitochondrial function, aiming to highlight their potential roles in the etiology of sporadic PD.

**TABLE 1:** *PARK* genes that are linked with mitochondrial function.

**Locus**	**Genes**	**Mutation**	**No. of cases reported**	**Mean age at onset**	**Disease progression/disease duration**	**Initial signs and symptoms (Top 5 whenever available)**	**L-Dopa response (out of PD patient tested)**	**α-synuclein pathology**	**Brain region M vs Y Up-regulated (≥2 folds)**	**Brain region M vs Y Down-regulated (≥2 folds)**
**Autosomal dominant inheritance**										
*PARK1/PARK4*	*SNCA*	Missense (A30P^, E46K^, H50Q^, G51D^, A53E^, A53T^, A32V^) or multiplication^^#^	146	40s	Rapid (<10 years)	Bradykinesia; Rigidity; Depression	Good (50/103)	Yes	ATZ, BLA, Pu	BMA, 10, RaM
*PARK8*	*LRRK2*	Missense (R1441G^, Y1699C^ G2019S, I2020T, G2385) or gain of function	724	50s	Rapid (15–20 years)	Tremor; Bradykinesia; Rigidity	Good (276/476)	Yes	CgGf,10, RaM, LC, Rpn, FuG	ATZ, BLA, BMA, Dt, CgGr, Rpn, Acb, VT
*PARK17*	*VPS35*	Missense (D620N^#^)	67	50s	Slow	Bradykinesia; Postural instability; Rigidity	Good (8/45)	Yes	BLA, Pu	BMA, PrG, RPN, FuG
**Autosomal recessive inheritance**										
*PARK2*	*PRKN*	Missense (K161N^#^, R256C^#^, R275W^, T415N^#^, 202–203 delAG^#^, 255delA^#^ and 321–322insGT^#^, W453STOP^#^), multiplication, deletion ^^#^, or loss of function	1,000	30s	Slow (27–50)	Tremor; Bradykinesia; Dystonia; Tremor at rest; Rigidity;	Good (192/427)	Yes	Dt	BMA, VTA
*PARK6*	*PINK1*	Missense (G411S^#^, Q456X^#^), deletion ^^#^ or loss of function	151	30s	Median (6–28)	Tremor; Bradykinesia; Rigidity; Dystonia; Tremor at rest	Good (84/113)	Yes	Dt,CA4,	ATZ, BMA, CgGr, SN,10, RaM, RPn, Pu, VT
*PARK7*	*DJ1*	Missense (A104T, M26I, L10P^#^, L166P^#^, L172Q^, and P159DEL^#^) or loss of function	33	20s	Slow	Bradykinesia; Dystonia; Tremor;	Good (5/25)	Yes	Crus II, CgGf, SN	BMA, CgGr, 10, RaM, RPn, Acb
**Atypical Parkinsonism**										
*PARK9*	*ATP13A2*	Missense (F182L, G504R, G877R, T12M^#^, G533R^#^, A746T^#^) or loss of function	36	10s	Slow	Bradykinesia; Intellectual development disorder; Cognitive decline; Gait difficulties; Rigidity	Good (9/30)	Yes (*in vitro*)	ATZ, BLA, BMA, CA4, VT	CgGf, CgGr, SPL, RPn, FuG, STG
*PARK14*	PLA2G6*	Missense (G31A, D331Y/M3581IfsX) or loss of function	7	20–30s	Rapid (1–15)	Bradykinesia; Rigidity; Spasticity; Hyperreflexia	Moderate	Yes	CgGf, CA2, SPL, Acb, Pu, FuG, STG	BMA, Dt, VTA, RaM, RPN,
*PARK15*	*FBXO7*	Missense (R378G^#^, R498X^#^, and T22M^#^)	26	10–20s	Rapid	Bradykinesia; Tremor; Gait difficulties; Rigidity; Behavioral abnormalities	Good (6/18)	Yes	CgGf	10, SPL, Pu, STG
*PARK22*	CHCHD2*	Missense (T61I^#^^)	19	50s	Long	Bradykinesia; Resting tremor; Posture instability;	Good	Yes	Dt, VTA, LC	BMA, PrG,10, SPL, RPn, Pu, FuG, STG
*PARK23*	*VPS13C*	Missense (A269S, W395C^, A444P^#^^, G1389R^#^^, Q1593L, and E3109STOP and deletion (V452-K3035)	4	20–30s	Rapid	Bradykinesia; Rigidity; Dystonia; Depression	Moderate	Yes	CgGf, VTA, LC	CgGr, SPL, RPn, Acb

*Information for *SNCA, LRRK2, VPS35, PRKN, PINK1, DJ1, ATP13A2, PLA2G6, FBXO7*, and *VPS13C* in this table were extracted from MDS gene, International Parkinson and Movement Disorder Society (http://msdgene.org). MDSGene currently collects data on 1651 different mutations in 6628 movement disorder patients extracted from 1250 publications (Klein et al., 2018). ^∗^Information for *PLA2G6 and CHCHD2* gene extracted from Online mendelian Inheritance in Man^®^ (http://omim.org). ^#^Mutations were shown to affect mitochondria function. ^Mutations were shown to involve in the formation of α-synuclein pathology. Abbreviations: Y-Young age (age of 24, 31, and 39), M-Middle age (age of 49, 55, and 57). M vs Y up-regulated: up-regulated gene expression with a fold change of greater or equal to 2. M vs Y down-regulated: down-regulated genes expression with a fold change of greater or equal to 2 (M vs Y).*

## *Park* Genes in Mitochondrial Function and Homeostatic Control

### *PARK1*/4 (*SNCA*): α-Synuclein

*SNCA* was the first *PARK* gene discovered to cause PD (Polymeropoulos et al., 1996). The prevalence of *SNCA* mutations is estimated as ∼0.05% in a cohort size of more than 2,000 sporadic PD patients (Tan et al., 2019). *SNCA* mutations can cause early-onset PD of variable clinical phenotypes and diverse Lewy pathologies (Campelo and Silva, 2017). Duplications or triplications of *SNCA* cause gene dosage effect on PD severity (Ibanez et al., 2004; Miller et al., 2004).

The physiological function of α-synuclein is not fully understood. Consistent with its many functions, this native disordered protein locates in multiple cellular organelles and sites: mitochondria, nucleus, synapse, endoplasmic reticulum (ER), Golgi, and lysosomes (Bernal-Conde et al., 2019; Shahmoradian et al., 2019).

α-Synuclein directly and indirectly interacts with mitochondria (Ganguly et al., 2018; Grunewald et al., 2019). It maintains mitochondrial membrane composition and structure and its deposition in neurons alters mitochondrial morphology and fragmentation, membrane potential, respiratory chain complex I function, and increases oxidative stress (Di Maio et al., 2016; Zambon et al., 2019; Hannestad et al., 2020). Overexpression of mitochondria-targeted α-synuclein results in mitochondrial reactive oxygen species (ROS) formation, reduced ATP levels, and neuronal death (Ganjam et al., 2019). *Vice versa*, mitochondrial dysfunction causes α-synuclein pathology as shown in traditional PD models induced by paraquat and rotenone. In addition, α-synuclein interacts with a number of critical mitochondrial proteins, including voltage-dependent anion-selective channel 1, PINK1, Parkin, and DJ-1 proteins (Bernal-Conde et al., 2019). It is also associated with mitochondrial Sirtuin 3, a nicotinamide adenine dinucleotide (NAD+)-dependent enzyme critical in mitochondrial quality control and the prevention of oxidative stress (Park et al., 2020).

### *PARK2* (*PRKN*): Parkin RBR E3 Ubiquitin Protein Ligase (*parkin*)

*PRKN*, the second identified PD gene (Matsumine et al., 1997) is the most common autosomal recessive gene causing early onset PD (Klein and Lohmann-Hedrich, 2007; Jiang et al., 2020). There are over 100 known mutations in *PRKN* that lead to either a dysfunctional small Parkin protein being rapidly degraded or defective parkin without activity (Abbas et al., 1999). Some, but not all *PRKN* mutations cases have Lewy Pathology (Farrer et al., 2001; Shimura et al., 2001; Pramstaller et al., 2005; Miyakawa et al., 2013; Johansen et al., 2018).

Parkin is a HECT/RING hybrid ligase that receives ubiquitin on its catalytic center and passes ubiquitin onto its substrates (Trempe et al., 2013). It regulates mitochondrial quality control through mitophagy and mitochondrial biogenesis. A loss of Parkin function contributes to the pathogenesis of PD through affecting mitochondria, innate immunity, and interactions with α-synuclein. In human cell models, the lack of *parkin* altered mitochondrial respiratory chain function, oxidative stress levels, mitochondrial morphology and motility, mitophagy (Koentjoro et al., 2017; Bonello et al., 2019), and mitochondrial biogenesis [by upregulating PARIS and subsequently downregulating PGC-1α (Kumar et al., 2020)]. Notably, loss of *Parkin* alone is not sufficient to induce dopaminergic (DA) neuron loss or motor deficits in mouse models (AguiarJr., Tristao et al., 2013). However, in combination with a *POLG* mutation (a proofreading-defective mtDNA polymerase), *Parkin*-deficient mice have both DA neuron loss and motor defects (Sliter et al., 2018).

### *PARK6* (*PINK1*): PTEN Induced Kinase 1 (*PINK1*)

*PINK1* is the second most common autosomal recessive gene for PD identified in 2004 (Valente et al., 2004). Heterozygous pathogenic mutations were found in both sporadic and familial PD (Klein et al., 2007). More than 70 mutations have been identified in *PINK1* (Siuda et al., 2014; Puschmann et al., 2017). Heterozygous G411S mutation cells have normal PINK1 levels but reduced kinase activity, altered ubiquitin phosphorylation, parkin recruitment, and mitophagy, whereas heterozygous Q456X mutation cells have reduced levels of PINK1 with decreased kinase activity, but their mitochondrial response to damage remains intact (Puschmann et al., 2017). Most but not all *PINK1* mutation cases have Lewy pathology, gliosis, and DA neuronal loss in the substantia nigra (Samaranch et al., 2010; Takanashi et al., 2016; Nybo et al., 2020).

PINK1 is a mitochondrial serine/threonine-protein kinase that recruits parkin to depolarized mitochondria for mitophagy (Matsuda et al., 2013). *Pink1*-deficient mice show significantly impaired mitochondrial respiration in the brain with aging, but no altered mitochondrial morphology, DA neuron loss, or Lewy pathology (Kitada et al., 2007; Gautier et al., 2008). Interestingly, *Pink1*-deficient rats exhibit DA neuron loss, altered neurotransmitters, and Lewy pathology at 12 months (Creed and Goldberg, 2018, 2020; Creed et al., 2019). Although PINK1/parkin are essential in the mitophagy pathway, they are not required in basal mitophagy (McWilliams et al., 2018). PINK1/parkin are significantly involved in regulating the basal inflammatory response (Sliter et al., 2018; Wang et al., 2019). Human cell models that lack PINK1 have altered mitochondrial respiratory chain function, morphology, motility, and mitophagy [reviewed in Grunewald et al. (2019)].

### *PARK7* (*DJ-1*): Parkinsonism Associated Deglycase (*DJ-1*)

Mutations in *DJ-1* were identified as a rare cause of early onset recessive PD in 2003 (Bonifati et al., 2003). Around 20 pathogenic *DJ-1* mutations have been identified with reduced protein due to rapid degradation (Ramsey and Giasson, 2010), and less dimerization into its functional form (Kumar et al., 2019). The autopsy of a patient with L172Q mutation showed severe DA neuronal loss in the substantia nigra with Lewy pathology (Taipa et al., 2016). About 57% of *DJ-1* mutation carriers exhibit non-motor symptoms, a higher proportion than *PRKN* or *PINK1* mutation carriers (Kasten et al., 2018).

DJ-1 is involved in cellular transformation, oxidative stress response, and mitochondrial function (Di Nottia et al., 2017; Raninga et al., 2017). DJ-1 responds to oxidative stress by accumulating on the outer mitochondrial membrane (OMM) in a PINK1/parkin dependent manner (Thomas et al., 2011; Joselin et al., 2012) which may be neuroprotective (Piston et al., 2017). Depletion of DJ-1 leads to increased ROS, decreased mitochondrial membrane potential, and accumulation of dysfunctional mitochondria, which can be rescued by increasing parkin (Andres-Mateos et al., 2007; Trempe and Fon, 2013; Ozawa et al., 2020). DJ-1 directly interacts with α-synuclein monomers and oligomers in mouse brains (Zondler et al., 2014) and DJ-1 deficiency increases α-synuclein aggregation in human and mouse models (Shendelman et al., 2004; Xu et al., 2017). Notably, the loss of DJ-1 does not induce nigral DA neuron demise in mice (Goldberg et al., 2005). *Dj-1*-deficient rats show DA neuron loss and evident motor abnormalities (Dave et al., 2014). Similar to *SNCA*, *LRRK2*, and *UCHL1*, mutations in *DJ-1* block or reduce the activity of chaperone-mediated autophagy (Sala et al., 2016).

### *PARK8* (*LRRK2*): Leucine-Rich Repeat Kinase 2 (*LRRK2*)

*LRRK2*, discovered in 2004, is the most frequent autosomal dominant gene causing PD with more than 100 mutations (Paisan-Ruiz et al., 2004; Rui et al., 2018). Genome Aggregation Database predicts LOF in *LRRK2* variants cause an 82.5% reduction in protein level, with no change in lifespan or clinical phenotype (Whiffin et al., 2020). *LRRK2* mutations generally develop later in life and are clinically similar to sporadic PD, although up to 50% do not have Lewy pathology. Mouse models studying G2019S, R1441G, and *Lrrk2*-deficient failed to show correlation between loss of function (LOF) of LRRK2 and α-synuclein pathology (Daher et al., 2012; Xiong et al., 2017) but impaired parkin-mediated mitophagy is found in fibroblasts from patients with the G2019S mutation (Bonello et al., 2019).

LRRK2 has multiple domains including a kinase and GTPase enzyme. It is involved in a wide range of cellular processes (Berwick et al., 2019; Marchand et al., 2020) and interacts with Miro on OMM to promote its removal, stopping mitochondrial motility and initiating mitophagy (Hsieh et al., 2016). RAB10, a substrate of LRRK2 kinase activity, accumulates on depolarized mitochondria and interacts with the autophagy receptor OPTN (optineurin) to mediate mitophagy in a PINK1/parkin-dependent manner (Wauters et al., 2020). The toxic gain in function of LRRK2 kinase activity inhibits the accumulation of RAB10 on mitochondria (Wauters et al., 2020). The lack of LRRK2 in macrophages induces oxidative stress and dynamin-related protein 1 (DRP1)-dependent mitochondrial fragmentation (Weindel et al., 2020).

### *PARK9* (*ATP13A2*): ATPase Cation Transporting 13A2 (*ATP13A2*)

Loss of function of *ATP13A2* was initially reported in Kufor-Rakeb syndrome (KRS; Ramirez et al., 2006) and in three other distinct neurodegenerative conditions: juvenile-onset neuronal ceroid lipofuscinosis (Bras et al., 2012), juvenile-onset hereditary spastic paraplegia (Estrada-Cuzcano et al., 2017), and amyotrophic lateral sclerosis-like phenotype (Spataro et al., 2019). More than 30 mutations have been identified in *ATP13A2* and rare variants may contribute to PD risk (Cristina et al., 2020). Mutations in *ATP13A2* cause decreased protein stability, increased proteasomal degradation, impaired polyamine transport and accumulation in lysosomes, and cell death (Podhajska et al., 2012; van Veen et al., 2020).

ATP13A2 is a lysosomal protein, located in the ER, endosomal and lysosomal membranes of neurons (de Tezanos Pinto and Adamo, 2018; Spataro et al., 2019). Loss of *ATP13A2* in mouse and human cell models increase mitochondrial fragmentation and increase ROS and cell death (Gusdon et al., 2012; Park et al., 2014). Cell models from KRS patients and cells with silenced *ATP13A2* show α-synuclein oxidation and accumulation (Tsunemi and Krainc, 2014). Over-expression of ATP13A2 reduces intracellular α-synuclein via the release of exosomes (Kong et al., 2014).

### *PARK14* (*PLA2G6*): Phospholipase A2 Group VI (*PLA2G6*)

*PLA2G6* mutations was discovered in a large family with neurodegeneration in 2006 (Morgan et al., 2006). Mutations in *PLA2G6* can cause autosomal recessive PD with high clinical variability, but all show cerebral and cerebellar atrophy, iron accumulation in the basal ganglia, cognitive decline (Khateeb et al., 2006; Ferese et al., 2018) and marked Lewy pathology (Paisan-Ruiz et al., 2012). A total of 16 mutations have been reported. Although most mutations of this gene are homozygous, a heterozygous missense mutation (G31A) has been reported to increase the risk of PD (Ferese et al., 2018). In contrast, compound heterozygous mutations (D331Y/M358IfsX) cause dystonia-parkinsonism with a poor response to levodopa (Chu et al., 2020).

PLA2G6, a calcium-independent phospholipase A2, is involved in maintaining mitochondrial function (Chiu et al., 2017). Overexpression of PLA2G6 exerted neuroprotection in human cells by increasing the level of mitophagy proteins in response to rotenone (Chiu et al., 2017). The loss of PLA2G6 results in shortened acyl-chains in phospholipids, which affects ER homeostasis, neurotransmission, and promotes α-synuclein aggregation (Mori et al., 2019). Elevated expression of α-synuclein in neuronal mitochondria is observed in PLA2G6 deficiency (Sumi-Akamaru et al., 2016).

### *PARK15* (*FBXO7*): F-Box Protein 7 (*FBXO7*)

A homozygous mutation in *FBXO7* was reported to cause autosomal recessive PD (Shojaee et al., 2008). Mutations in *FBXO7* have not been detected in sporadic PD (Conedera et al., 2016). Mutations in *FBXO7* promote the aggregation of the toxic form of this protein in mitochondria, resulting in impairment of mitophagy and the ubiquitin-proteasome system (Zhou et al., 2015). *FBXO7* mutations and *SNCA* G51D mutation have been implicated in Parkinsonian-pyramidal syndrome with early onset and rapid progression (Joseph et al., 2018).

FBXO7 is an adaptor protein in Skp-Cullin-F-box (SCF) SCF^*FBXO*7^ ubiquitin E3 ligase complex, which recognizes substrates and mediates their ubiquitination and translocation to mitochondria following cellular stress (Winston et al., 1999; Joseph et al., 2018). FBXO7 recruits parkin into damaged mitochondria and facilitates its aggregation, but overexpression of FBXO7 can still rescue DA neuron degeneration in parkin null Drosophila (Burchell et al., 2013; Zhou et al., 2016) and restore PD phenotype in the absence of parkin, indicating FBXO7 mediates neuroprotective effects via a parkin-independent pathway (Burchell et al., 2013). Both soluble and insoluble FBXO7 are increased in PD (Zhou et al., 2015). FBXO7 immunoreactivity is detected in most α-synuclein aggregates in PD and in glial cytoplasmic inclusions of multiple system atrophy (Zhao et al., 2013). In contrast, only occasional tau-positive inclusions in Alzheimer’s disease and progressive supranuclear palsy contain FBXO7.

### *PARK17* (*VPS35*): Vacuolar Protein Sorting 35 Ortholog (*VPS35*)

Mutations in *VPS35* were identified in 2008 (Wider et al., 2008) and are reported in patients with autosomal dominant PD. A heterozygous missense mutation D620N has been confirmed as pathogenic (Williams et al., 2017; Chen et al., 2019) and has been found in 0.056∼0.91% of the sporadic PD patients (Ando et al., 2012; Kumar et al., 2012). The D620N mutation did not affect the stability, assembly, or subcellular location of the retromer (Tian et al., 2015), instead it enhanced LRRK2 kinase activity (Mir et al., 2018). D620N mutant mice show no motor disorders but have increased mitochondrial fission and fragmentation (Wang et al., 2017; Cataldi et al., 2018).

VPS35 forms part of a retromer cargo-recognition complex involved in intracellular retrograde transport from endosomes to the *trans*-Golgi network (Hierro et al., 2007; Tabuchi et al., 2010). Loss of iPLA2-VIA (the Drosophila homolog of human PLAG2A) destabilizes VPS35 and impairs retromer function, resulting in ceramide accumulation and cell stress (Lin et al., 2018). VPS35 is implicated in the formation of mitochondria-derived vesicles directed to the peroxisome or lysosome for degradation of mitochondria proteins (Braschi et al., 2010; Wang et al., 2016). Lack of VPS35 in human cells with *VPS35* mutations exhibit defective mitochondrial fusion and increased mitochondrial fragmentation (Tang et al., 2015). Mitochondrial dysfunction induced by *VPS35* mutation can be restored by inhibition of mitochondrial fission (Wang et al., 2016). α-Synuclein is transported by the retromer complex (Miura et al., 2014). Heterozygous *Vps35* KO mice show α-synuclein aggregation, DA neuron degeneration, impaired locomotor behavior, and altered lysosomal morphology (Tang et al., 2015). Overexpression of VPS35 reduces α-synuclein accumulation in mice overexpressing α-synuclein (Dhungel et al., 2015). Moreover, knockdown *Vps35* in Drosophila results in α-synuclein accumulation (Miura et al., 2014).

### *PARK22* (*CHCHD2*): Coiled-Coil-Helix-Coiled-Coil-Helix Domain Containing 2 (*CHCHD2*)

Mutations in *CHCHD2* are a rare cause of autosomal dominant PD, originally found in 3/340 PD patients in 2015 (Funayama et al., 2015). To date, there is only one brain autopsy of a PD patient carrying *CHCHD2* T61I mutation that revealed widespread Lewy pathology with additional amyloid plaques and neurofibrillary tangles in the brainstem, limbic regions, and neocortex (Ikeda et al., 2019). α-Synuclein aggregation was accelerated by *CHCHD2* T61I in human cells and in Drosophila. Human cells from T61I patients show accumulated CHCHD2 in the mitochondrial intermembrane space (IMS), resulting in increased ROS and apoptosis (Cornelissen et al., 2020).

CHCHD2 (also called mitochondria nuclear retrograde regulator 1) contains at least one CHCH domain (Modjtahedi et al., 2016). The protein locates in the IMS (Aras et al., 2015). Loss of CHCHD2 function causes an abnormal mitochondrial matrix structure and impaired oxygen respiration in mitochondria resulting in oxidative stress, DA neuron loss, and motor dysfunction with aging (Meng et al., 2017). Importantly, overexpression of CHCHD2 rescues the phenotype of PD. In addition, CHCHD2 binds to cytochrome *c* and Bax inhibitor-1, suggesting the role CHCHD2 in regulating apoptosis and cell death (Liu et al., 2015). In Drosophila, CHCHD2 interacts with the mitochondrial protein P32 and indirectly regulates the level of mitochondrial fusion protein, Opa1, highlighting the role of CHCHD2 in regulating mitochondrial fusion and cristae morphology (Liu et al., 2015). Moreover, human cells lacking CHCHD2 have altered mitochondrial respiration (Harjuhaahto et al., 2020).

### *PARK23* (*VPS13C*): Vacuolar Protein Sorting-Associate Protein 13C

Mutations in *VPS13C*, identified in 2016 cause an autosomal recessive early onset PD, characterized by early cognitive decline and rapid disease progression (Lesage et al., 2016; Schormair et al., 2018). The post-mortem examination of the brain of the affected patient displayed reduced protein levels of VSP13C and the presence of α-synuclein pathology (Lesage et al., 2016; Smolders et al., 2021).

VPS13C acts at membrane contact sites on multiple organelles such ER, mitochondria, and late endosome and lysosome for lipid delivery, which is important for mitochondrial biogenesis and function (Kumar et al., 2018). VPS13C was localized to the OMM as shown in HEK293 cells (Lesage et al., 2016) and was found between lipid droplets and mitochondria (Ramseyer et al., 2018). LOF in VPS13C in COS-7 monkey cells resulted in abnormal mitochondrial morphology, increased vulnerability to stress and the activation of PINK1/parkin-dependent mitophagy (Lesage et al., 2016). Overexpression of W395C or A444P VPS13C in Hela or SH-SY5Y cells showed the ER-endosomal/lysosomal localization of VPS13C was lost, suggesting these mutants might affect the stability of the protein thereby influencing its localization (Smolders et al., 2021).

## Discussion

Over the past 20 years, great progress has been in our understanding of PD with the identification of 23 *PARK* genes. No doubt there will be more that await discovery. The 11 *PARK* genes highlighted here collectively emphasize the mechanistic importance of mitochondrial function underlying that pathobiology of PD. These genes are involved in multiple pathways affecting mitochondrial morphology, quality control, respiratory chain function, release of ROS, and biogenesis (fission/fragmentation). More importantly, proteins encoded by five genes (*PRKN, PINK1, DJ-1*, *LRRK2*, and *FBXO7*) closely interact with α-synuclein. Mutations in *LRRK2, ATP13A2, PLA2G6, VPS35, CHCHD2*, and *VPS13C* lead to increased α-synuclein accumulation, and mutations in *SNCA*, *PRKN, PINK1, DJ-1*, *LRRK2*, and *VPS35* are responsible for the loss of DA neurons.

**FIGURE 1:**
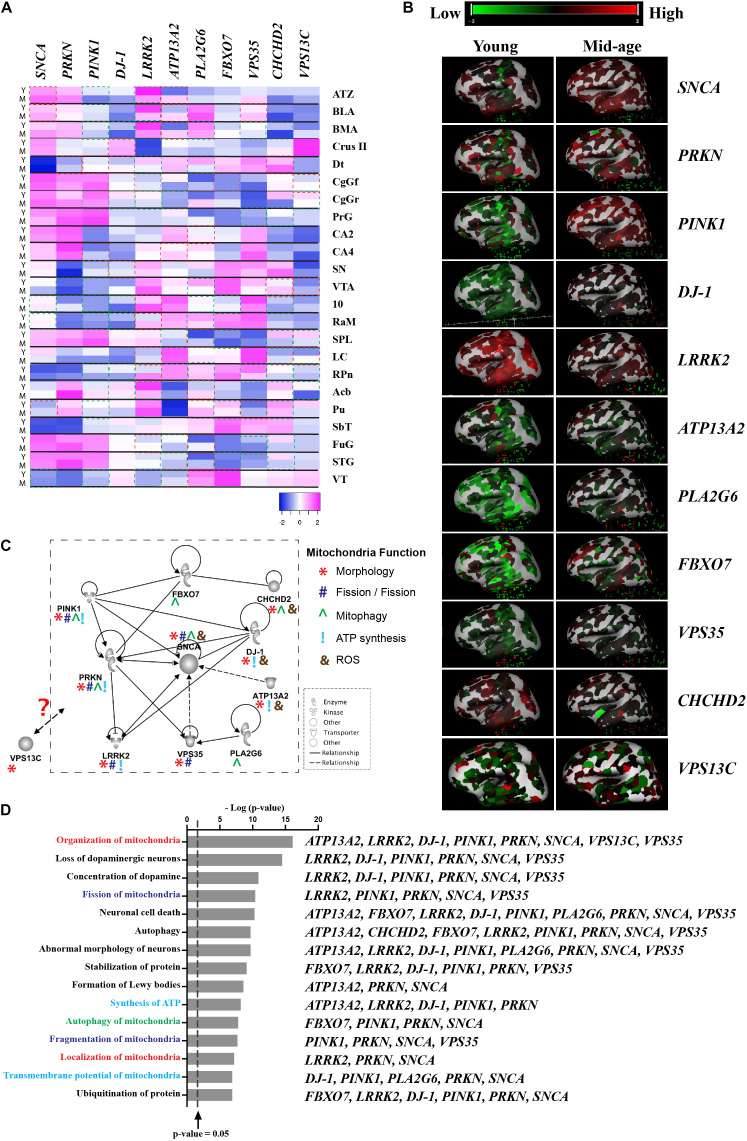
Interaction of proteins encoded by mitochondrial-related *PARK* genes. **(A)** Gene expression data was obtained from Allen Brain Atlas, Allen Institute. *n* = 2–3/group. Y-Young (age of 24, 31, and 39), M-Middle age (age of 49, 55, and 57). Heatmap showing the z-score was generated using online Heatmap program (Babicki et al., 2016). Red boxes indicates up-regulated gene expression with a fold change of greater or equal to 2 (M vs Y) and green boxes indicates down-regulated genes expression with a fold change of greater or equal to 2 (M vs Y). **(B)** 3D heatmap of the 11 genes from Allen Brain Atlas, Allen Institute. Young (age of 24) and mid-age (age of 57); **(C)** The interactive pathways of proteins encoded by the 11 *PARK* genes generated from IPA (Ingenuity Systems Inc., Redwood city, CA, United States). Symbols are used to show mitochondrial function: *Morphology; ^#^Fission/Fusion; ^Mitophagy; ^!^ATP synthesis; and ^&^ROS. **(D)** Top 35 biological functions identified to be related to mitochondrial function and DA neuron survival and relevant *PARK* genes listed next to functions. The biological functions were plotted against the negative log10 *p*-value [–log(*p*-value)] as measured by Fischer’s exact test determined by IPA. Line represented *p*-value = 0.05. Abbreviations: ATZ, amygdalohippocampal transition zone; BLA, basolateral nucleus; BMA, basomedial nucleus; Cb-Crus II, crus II; Dt, dentate nucleus; CgGf, cingulate gyrus; frontal part; CgGr, cingulate gyrus, retrosplenial part; PrG, precentral gyrus; CA2, CA2 field; CA4, CA4 field; SN, substantia nigra; VTA, ventral tegmental area; 10, dorsal motor nucleus of the vagus; RaM, raphe nuclei of medulla; SPL, superior parietal lobule; LC, locus ceruleus; RPn, pontine raphe nucleus; Acb, nucleus accumbens; Pu, putamen; SbT, subthalamus; FuG, fusiform gyrus; STG, superior temporal gyrus; and VT, ventral thalamus.

Although there is a lack of topographical mapping of these 11 gene coding proteins, the heatmap of their RNA expression is available for the human brain (Figures 1A,B; ^©^ 2010 Allen Institute for Brain Science. Allen Human Brain Atlas. Available from: human.brain-map.org), suggesting *PARK* gene expression is both age and brain region related, which further highlight regional vulnerability in the profiling of these proteins. Assessment of pathways affected by these 11 *PARK* genes using Ingenuity^®^ Pathway Analysis software (Ingenuity Systems Inc., Redwood city, CA, United States) reveals links to DA neuron survival, mitochondrial function, formation of Lewy body pathology, and their mutual protein interactions (Figures 1C,D). Hitherto, cell type specific expression of these gene coding proteins remains unknown. This review suggests the knowledge gap in the field and highlights the importance of studying these genes in sporadic PD, which is essential before targeting these mitochondrial pathways for disease modification.

## Author Contributions

WL and YF conceived of the presented data. WL and YF wrote the manuscript in consultation with GH and CS. GH and CS were in charge of overall direction and planning. All authors contributed to the article and approved the submitted version.

## Conflict of Interest

The authors declare that the research was conducted in the absence of any commercial or financial relationships that could be construed as a potential conflict of interest.
